# The Occurrence of Bacterial Infections in Equine Wounds and Abscesses in Horses from 2019 to 2023

**DOI:** 10.3390/vetsci13060584

**Published:** 2026-06-16

**Authors:** Justyna Kłopotowska, Eva Maria Kalbhenn, Babette Klein, Sabita Diana Stöckle, Roswitha Merle, Elisabeth Müller, Heidrun Gehlen

**Affiliations:** 1 Department of Internal Medicine, Equine Clinic, Freie Universität Berlin, Oertzenweg 19b, 14163 Berlin, Germany; justyna.klopotowska@fu-berlin.de (J.K.); s.stoeckle@fu-berlin.de (S.D.S.); 2 Phage Center, Sieboldstraße 7, 97688 Bad Kissingen, Germany; kalbhenn@laboklin.com (E.M.K.); b.klein@laboklin.com (B.K.); 3 LABOKLIN GmbH & Co. KG, Steubenstraße 4, 97688 Bad Kissingen, Germany; mueller@laboklin.com; 4 Institute of Veterinary Epidemiology and Biostatistics, Freie Universität Berlin, Königsweg 67, 14163 Berlin, Germany; roswitha.merle@fu-berlin.de

**Keywords:** wound infections, horses, Germany, MRSA, 3/4 GCR *Escherichia coli*, retrospective study

## Abstract

Bacterial infections in equine wounds are common and can complicate treatment and healing. This five-year retrospective study examined 2844 samples from wounds and abscesses from horses in Germany (2019–2023), analyzing bacterial prevalence by care setting and geographical region. A total of 4464 bacterial isolates were identified, with β-hemolytic streptococci (20.6%), *Escherichia coli* (15.6%), and *Staphylococcus aureus* (13.0%) being the most common. Methicillin-resistant *Staphylococcus aureus* (MRSA) accounted for 35.9% of *Staphylococcus aureus* isolates. Resistance to third- and fourth-generation cephalosporins was detected in 15.2% of *Escherichia coli* isolates. Samples from clinics showed a significantly higher MRSA rate, while primary care samples more often contained environmental bacteria. Regional differences in pathogen prevalence and statistically significant temporal changes were observed. The results highlight the importance of continuous microbiological surveillance and underscore the need for targeted antimicrobial stewardship in veterinary facilities. These findings have implications for infection control, empirical treatment strategies, and zoonotic risk mitigation in equine medicine.

## Simple Summary

Wounds and abscesses in horses often become infected with bacteria, which can prolong healing and cause serious health problems. Over 2800 wound and abscess samples collected from horses in Germany between 2019 and 2023 were analyzed in this study to determine the most common bacteria and the bacterial occurrence over time, across regions, and between home-based primary care and clinical settings. The main bacterial groups identified were ß-hemolytic streptococci, *Escherichia coli*, and *Staphylococcus aureus*, with methicillin-resistant *Staphylococcus aureus* occurring more frequently in samples from clinics. The study also revealed regional differences in bacterial species/groups and identified increasing or decreasing changes. These findings help veterinarians to choose more effective treatments, avoid unnecessary antibiotics, and protect the well-being of horses and humans in the manner of One Health.

## 1. Introduction

Bacterial infections are among the most common complications associated with wounds and abscesses in horses. They often delay healing, prolong treatment, and increase the animal’s suffering. If left untreated, wound infections can lead to chronic pain and impair performance and welfare, partly through excessive granulation tissue (“proud flesh”) [1,2], which also affects esthetics. The horse’s condition may deteriorate in severe cases and necessitate euthanasia [3]. Identifying the causative organisms and understanding their antimicrobial susceptibility profiles is, therefore, crucial for selecting appropriate empirical and targeted therapies.

Wound infections are defined as the presence of proliferating microorganisms in a wound that cause tissue damage and harm the host [4]. The presence of bacteria alone does not necessarily indicate infection; the latter occurs when one or more pathogens evade the host’s defenses, proliferate, and damage tissue [4]. The microbial composition of wounds differs depending on the wound depth, with deeper layers often harboring a more diverse flora [4]. Abscesses typically develop after puncture wounds from injections, injuries, or the penetration of foreign bodies.

Recent studies [5,6] have identified *Enterobacteriaceae*, including *Escherichia* (*E.*) *coli*, *Streptococcus* spp., and *Staphylococcus* (*S.*) spp., as the predominant pathogens causing equine wound infections. Due to the close contact of horses with humans and their role in sport and therapy, horses can act as reservoirs of resistant pathogens, thus contributing to zoonotic transmission [7]. Pathogenic bacteria are not only a concern for equine patients, but pose a risk to veterinarians and animal care workers [8]. These professionals face an increased risk of colonization and infection with methicillin-resistant *Staphylococcus aureus* (MRSA) [9]. Several reports have highlighted increased MRSA detection in horses in various countries, for example, Canada, the USA, the UK, and West Europe [10,11,12,13]. The prevalence of bacteria can vary depending on the type of wound, breed (horse or pony), anatomical location, environmental influence, the presence of biofilm, and possibly age, although its role remains less clearly defined [14,15,16,17,18].

However, there is still a lack of long-term, large-scale studies investigating temporal changes in the bacterial distribution in equine wounds, particularly regarding differences between types of veterinary care and geographic regions. Although previous studies have addressed microbial dynamics, including biofilm formation and antimicrobial resistance (AMR) patterns [19,20,21], comprehensive analyses integrating regional variation with differences between clinical settings remain limited. This gap is particularly evident in Germany, where comparative retrospective microbiological data across different types of veterinary care are scarce. At the same time, the increasing prevalence of multidrug-resistant strains, especially MRSA, and extended-spectrum beta-lactamase (ESBL)-producing Enterobacteriaceae, including *E. coli*, poses a growing challenge for equine wound management and underscores the need for up-to-date surveillance data [1,22]. This development complicates empirical antimicrobial therapy and highlights the growing importance of antimicrobial stewardship strategies in equine medicine. Culture-based diagnostics, targeted antimicrobial use, and surveillance of regional resistance patterns are increasingly recognized as essential components of responsible antimicrobial prescribing and preservation of antimicrobial efficacy [22].

The main objective of this study was to characterize the most prevalent bacterial species in equine wounds and abscesses from horses over a five-year period, assess temporal changes in their prevalence, and evaluate differences between primary and clinic-based care as well as variations in geographical regions. It is hypothesized that the distribution of certain pathogen groups changes significantly over time, the microbial profiles differ between primary care and clinics, and geographical variation plays a substantial role in the prevalence of pathogens.

## 2. Materials and Methods

This study presents a five-year retrospective analysis of bacterial isolates from wounds and abscesses in horses, integrating temporal, regional, and care-level types to support clinical decision-making and antimicrobial stewardship in equine medicine.

Anonymized diagnostic data were supplied by the accredited veterinary diagnostic laboratory Laboklin GmbH & Co. KG (Bad Kissingen, Germany) and analyzed retrospectively. The data originated from bacterial samples from wounds and abscesses of horses sent in from veterinarians in Germany.

All samples were submitted for routine diagnostic purposes; therefore, no consent form or ethical approval was required. All 2844 samples labeled as wounds or abscesses by veterinarians between 2019 and 2023 were included. Samples from abscesses of the oral cavity or other non-cutaneous sites were excluded.

### 2.1. Microbiological Analysis

The microbiological examinations were performed at the accredited veterinary diagnostic laboratory Laboklin GmbH & Co. KG (Bad Kissingen, Germany), following the protocols described by Schwab et al. [23] and Leps et al. [24].

### 2.2. Bacterial Culture and Identification

Swabs were streaked onto Columbia Agar with 5% sheep blood (Becton Dickinson GmbH, Heidelberg, Germany/Oxoid GmbH, Wesel, Germany) and Endo Agar (Becton Dickinson, Heidelberg, Germany). The plates were incubated at 36 °C (±1 °C) under aerobic conditions overnight. After 18–24 h, the plates were examined for bacterial growth. If no growth was observed, incubation was extended for an additional 18–24 h.

Additionally, the swabs were enriched by inoculation into Casein-Soy-Bean-Digest broth (Becton Dickinson GmbH, Heidelberg, Germany) and incubated overnight at 36 °C (±1 °C) under aerobic conditions. Afterwards, the enrichment broth was streaked onto Columbia Agar supplemented with 5% sheep blood and Endo Agar. The plates were then incubated aerobically overnight at 36 °C (±1 °C). Following incubation, bacterial growth was assessed after the enrichment step.

Bacterial species were identified based on growth characteristics on selective agar plates and biochemical tests, including MAST ID oxidase test strips [Diagnostica GmbH, Reinfeld, Germany], catalase [3% solution], latex agglutination [DiaMondial Staph Plus Kit, Langenhagen, Germany], and matrix-assisted laser desorption/ionization time-of-flight mass spectrometry (Bruker Daltonics, Bremen, Germany). All *Staphylococcus* spp. isolates were tested for methicillin resistance by an antimicrobial sensitivity test using the Micronaut system in accordance with the Clinical Laboratory Standards Institute guidelines for checking oxacillin or cefoxitin resistance [25]. The resistance to ceftiofur and cefquinome in the antimicrobial sensitivity test was used to determine the resistance to third- and fourth-generation cephalosporins (3/4 GCR) in *E. coli*.

### 2.3. Data Processing

The following information was extracted for each sample for the retrospective analysis: the date of collection, the first two digits of the postal code numbers indicating the geographical location within Germany, the sample identification number, the place of sampling (primary care [P] or veterinary clinics [C]), the sampling site (wounds or abscesses), and the bacterial species identified.

Data concerning prior antimicrobial treatment or the precise anatomical location of the wound or abscess were not available. Similarly, information regarding the hospitalization history and repeated sampling from the same horse could not be assessed due to the retrospective and anonymized nature of the laboratory dataset. All samples were anonymized regarding the submitting institution and the owner of the horse to ensure compliance with data protection regulations. The only information retained was the type of veterinary care in which the sample had been collected, categorized as either “clinic/hospital” (C) or “primary care” (P). This retrospective study was based on routine diagnostic submissions; therefore, standardized sampling protocols and detailed clinical information were not available. Consequently, differentiation between true infection, colonization, and possible sample contamination was not feasible on an individual case basis.

Each bacterial isolate recovered from a sample was treated as an individual analytical unit. Consequently, polymicrobial samples contributed more than one isolate to isolate-based analyses, and multiple isolates originating from the same sample were included as separate observations.

In this study, classification into P or C was based solely on the type of submitting veterinary institution recorded in the diagnostic database. The P referred to samples submitted by ambulatory veterinary practitioners, typically collected at the horse’s residence (e.g., stable or paddock), whereas C referred to samples submitted by equine clinics or hospitals. Information regarding the disease stage, previous treatment, referral status, or whether samples represented initial or follow-up examinations was not available.

Germany was meticulously divided into three regions to assess any geographical disparities in bacterial prevalence: the South (comprising Saxony, Thuringia, Hesse, Rhineland-Palatinate, Saarland, Baden-Württemberg, and Bavaria), the Northwest (including North Rhine-Westphalia, Lower Saxony, Bremen, Hamburg, and Schleswig-Holstein), and the Northeast (encompassing Mecklenburg-Western Pomerania, Saxony-Anhalt, Brandenburg, and Berlin).

### 2.4. Statistical Analysis

Descriptive data analyses were conducted using Microsoft^®^ Excel (Microsoft^®^ Excel, Version 2411, Microsoft Corporation, Redmond, WA, USA) and SPSS (Version 29.0.0, IBM, Armonk, NY, USA), as well as Python (Version 3.11.13) coding in Google Collaboratory (Google LLC, Mountain View, CA, USA), a cloud-based Jupyter notebook environment that provides collaborative and GPU-accelerated computing capabilities. The graphical data visualizations were generated using PowerPoint (Microsoft^®^ PowerPoint, Version 2411, Microsoft Corporation).

Chi-square (χ^2^) tests were employed to compare the distribution of bacterial isolates between types of veterinary care (C vs. P), across different geographical regions (South, Northeast, Northwest), and over time (2019–2023). Negative binomial regression models were applied to investigate temporal changes in the number of isolates for selected bacterial species. The annual number of isolates was designated as the numerator, whereas the total number of samples submitted each year was utilized as the denominator.

The calculation of proportions was conducted with 95% Wilson confidence intervals (95% CI) for various bacterial subgroups, for example, environmental bacteria and bacterial species. Subsequently, the variation in the prevalence of bacterial isolates between C and P was subjected to further evaluation employing odds ratios (OR) with 95% CIs derived from 2 × 2 contingency tables. An OR greater than 1 was interpreted as a higher chance of isolation of the species considered in samples obtained from veterinary clinics.

A two-step statistical approach was utilized. Initially, a χ^2^ test (2 × 3 contingency table) was conducted for each bacterial species to ascertain whether there were statistically significant differences in prevalence among the three regions. Notwithstanding the result of the overall test, pairwise post hoc comparisons were performed for all regional pairs using χ^2^ tests on 2 × 2 contingency tables. Bonferroni correction was applied for multiple comparisons. Results were considered statistically significant at a *p*-value < 0.05.

## 3. Results

A total of 2844 samples from wounds and abscesses were collected, from which 4464 bacterial isolates were obtained (Table 1). The proportion of positive samples to the total number of samples per year remained relatively stable from 2019 to 2023, ranging between 90.4 and 94.3%. The number of samples from South Germany (n = 1181) and Northwest Germany (n = 1074) was similar, in contrast to Northeast Germany, where only 373 positive samples were identified. Approximately twice as many samples originated from C (n = 1708) as from P (n = 786). No data was available regarding the assignment to C or P for 228 samples, resulting in only 4.1 to 7.5% (2019–2023).

The bacterial species isolated from equine wounds and abscesses were grouped as shown in Table 2. Isolates that could not be clearly assigned to main taxonomic groups were categorized into collective groups, such as other *Enterobacterales*, other Streptococci, other Staphylococci, and other Gram-negative bacteria. These categories comprise less frequently detected or taxonomically heterogeneous species that did not meet the criteria for separate listing but are summarized to ensure a structured and comprehensive presentation of the data.

### 3.1. Qualitative Analysis

**Figure 1:**
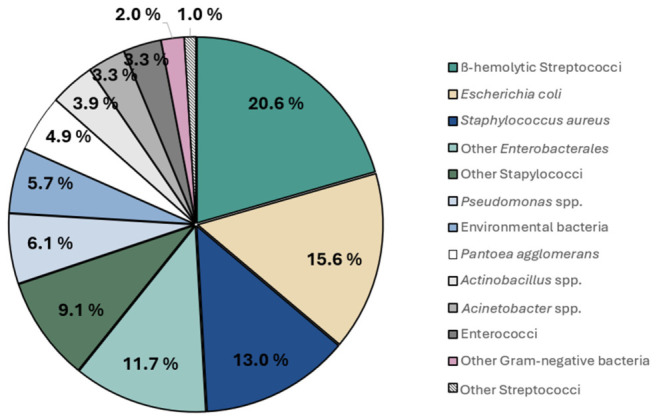
Distribution in percentage (%) of all bacteria isolated from wounds and abscesses of horses from 2019 to 2023.

A total of 4464 different bacteria were isolated from 2844 samples. The most prevalent bacterial group among all isolates was *ß*-hemolytic streptococci (n = 921; 20.6%), followed by *E. coli* (n = 696; 15.6%) and *S. aureus* (n = 579; 13.0%) (Figure 1). Other *Enterobacterales* accounted for 11.7% of all isolates (n = 520), while other staphylococci were present in 9.1% (n = 407) and *Pseudomonas* spp. in 6.1% (n = 270). Environmental bacteria represented 5.7% of the isolates (n = 252).

Less frequently identified species included *P. agglomerans* (n = 217; 4.9%), *Actinobacillus* spp. (n = 173; 3.9%), *Acinetobacter* spp. (n = 146; 3.3%), enterococci (n = 148; 3.3%), other Gram-negative bacteria (n = 89; 2.0%) and other streptococci (n = 46; 1.0%).

The MRSA occurred in 7.9% of positive samples and 3/4 GCR *E. coli* in 4.0%. The MRSA was identified in 208 isolates, accounting for 35.9% of all *S. aureus* isolates and 4.7% of all bacterial isolates. The 3/4 GCR *E. coli* was detected in 106 cases, representing 15.2% of all *E. coli* isolates and 2.4% of the total number of isolates.

### 3.2. Temporal Changes in Bacterial Occurrence

**Figure 2:**
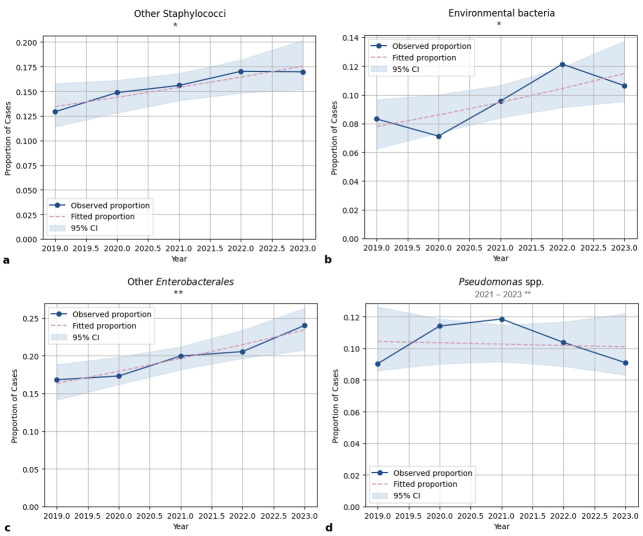
Overview of proportions of different bacteria observed over the years (2019–2023) of horses from wounds and abscesses including 95% confidence intervals (CIs) from regression models (* *p*-value < 0.05, ** *p*-value < 0.01) (**a**) Proportion of *Staphylococcus* spp. (**b**) Proportion of environmental bacteria. (**c**) Proportion of other *Enterobacterales*. (**d**) Proportion of *Pseudomonas* spp.

Several bacterial groups isolated from equine wounds and abscesses showed significant temporal changes in prevalence over the five-year study period. According to the negative binomial regression analyses, the prevalence of other staphylococci (*p*-value = 0.034), other *Enterobacterales* (*p*-value = 0.001), and environmental bacteria (*p*-value = 0.019) increased significantly over time (Figure 2), whereas *Pseudomonas* spp. (*p*-value = 0.0285) showed a significant decrease between 2021 and 2023. No significant temporal changes were identified for the remaining species or bacterial groups.

### 3.3. Differences Between C and P

Of the 2630 positive samples taken between 2019 and 2023, 1708 (64.9%) samples originated from C and 786 (29.9%) from P (Table 1). The origin of the remaining 136 samples (5.2%) could not be determined. Among C, 90.9% tested positive. In P, 95.6% yielded positive results (Table 3). Thus, no statistically significant difference was observed between the type of veterinary care concerning the percentage of positive results.

**Figure 3:**
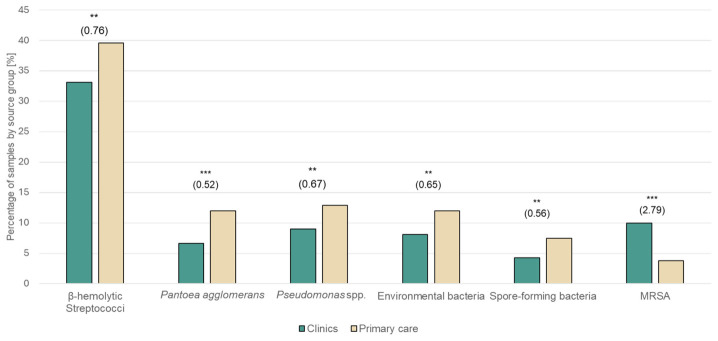
Distribution of bacterial groups isolated from equine wounds and abscesses collected between 2019 and 2023 comparing C and P. Only bacterial groups and species showing statistically significant differences are shown. The overview contains the prevalence percentages (%), *p*-values, and OR indicating the likelihood of occurrence in one facility (C or P) compared to the other (** *p*-value < 0.01, *** *p*-value < 0.001; χ^2^ test).

According to a χ^2^ test and OR analysis, the prevalence of certain bacterial groups varied significantly between C and P during the five-year period (Figure 3). The MRSA was significantly more prevalent in C (OR = 2.79, CI 1.87–4.15; *p*-value < 0.001). By contrast, β-hemolytic streptococci (OR = 0.76, CI 0.64–0.90; *p*-value = 0.002), *P. agglomerans* (OR = 0.52, CI 0.39–0.70; *p*-value < 0.001), *Pseudomonas* spp. (OR = 0.67, CI 0.51–0.88; *p*-value = 0.003), environmental bacteria (OR = 0.65, CI 0.49–0.86; *p*-value = 0.002), and the spore-forming bacteria (OR = 0.56, CI 0.39–0.79; *p*-value = 0.001) were significantly more common in P.

**Figure 4:**
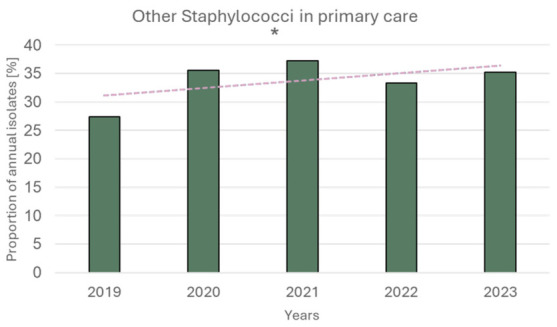
Proportion of other staphylococcal isolates among all bacterial isolates in P samples between 2019 and 2023. A statistically significant temporal change was observed over the five-year period, with a noticeable upward trend in the occurrence of this bacterial group (linear regression: slope = 2.7, * *p*-value = 0.0115, R^2^ = 0.9113). The dashed line represents the fitted proportion estimated by the regression model.

Temporal changes in the prevalence of bacterial groups and species were also analyzed separately for C and P. While no statistically significant temporal trends were detected for the majority of bacterial groups, an upward trajectory in the occurrence of the other staphylococci group was observed in P (slope = 2.7000, *p*-value = 0.0115, R^2^ = 0.9113) (Figure 4).

### 3.4. Regional Differences

**Figure 5:**
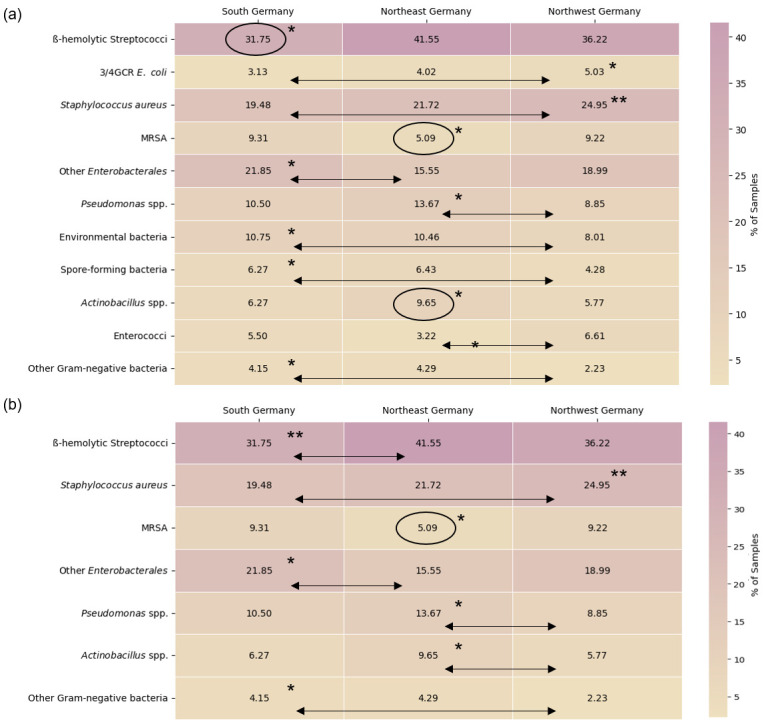
(**a**) Relative frequency (% of positive swabs) of bacterial groups and species identified in three German regions between 2019 and 2023 with unadjusted *p*-values. Only those showing statistically significant differences are presented. Rows represent bacterial species or groups, and columns correspond to geographic regions (South Germany, Northeast Germany, Northwest Germany). Color intensity reflects the proportion of isolates per region, normalized by the number of positive swabs collected in each region. Arrows indicate the specific regional comparisons in which statistically significant differences were observed. Ellipses highlight the region that differed significantly from both remaining regions. Statistical significance is indicated by asterisks (* *p*-value < 0.05, ** *p*-value < 0.01; χ^2^ test). (**b**) Relative frequency (% of positive swabs) of bacterial groups and species identified in three German regions between 2019 and 2023 after Bonferroni correction. For a detailed description, see (**a**).

The prevalence of bacterial groups and species varied significantly across the three regions (South Germany, Northeast Germany, Northwest Germany) over the five-year study period (2019–2023; χ^2^ tests, *p*-value < 0.05). The figure below represents the frequency of identified bacteria in each region without adjustment for multiple comparisions (Figure 5a) and after Bonferroni correction (Figure 5b). Both unadjusted and Bonferroni-adjusted *p*-values are reported; unadjusted *p*-values are presented first, followed by adjusted values in parentheses (*p*-value_adj).

The most prevalent group in all regions was β-hemolytic streptococci, with statistically significant differences among them (χ^2^ test, *p*-value = 0.0013). This represents the global comparison across all regions, while the results below refer to pairwise comparisons. Specifically, the South of Germany had significantly fewer positive observations of β-hemolytic streptococci than the Northeast (pairwise comparison, *p*-value = 0.0006; *p*-value_adj = 0.0019) and Northwest (*p*-value = 0.0282; *p*-value_adj = 0.0847). The 3/4 GCR *E. coli* (*p*-value = 0.0295; *p*-value_adj = 0.0885) and *S. aureus* (*p*-value = 0.0021; *p*-value_adj = 0.0062) were significantly more prevalent in the Northwest, while MRSA was significantly more common in the South (*p*-value = 0.0136; *p*-value_adj = 0.0408) and the Northwest (*p*-value = 0.0165; *p*-value_adj = 0.0495). Other *Enterobacterales* (*p*-value = 0.0105; *p*-value_adj = 0.0314) were more common in the South than the Northeast. The *Pseudomonas* spp. group (*p*-value = 0.0103; *p*-value_adj = 0.0308) was significantly more common in the Northeast than in the Northwest, whereas environmental bacteria (*p*-value = 0.0312; *p*-value_adj = 0.0935) were more common in the South. The same trend was observed for the spore-forming bacteria (*p*-value = 0.0454; *p*-value_adj = 0.136). *Actinobacillus* spp. were more prevalent in the Northeast compared to the South (*p*-value = 0.0351; *p*-value_adj = 0.1054) and Northwest (*p*-value = 0.0143; *p*-value_adj = 0.0430). Finally, enterococci (*p*-value = 0.0215; *p*-value_adj = 0.0645) occurred more frequently in the Northwest compared to the Northeast, and other Gram-negative bacteria (*p*-value = 0.0144; *p*-value_adj = 0.0433) showed higher prevalence in the South.

## 4. Discussion

To the best of our knowledge, this study represents one of the first large-scale retrospective analyses providing a comprehensive, five-year overview of bacterial species isolated from equine wounds and abscesses across different type of veterinary care settings and geographic regions in Germany (2019–2023). In this context, previous studies have mainly focused on individual pathogens or resistance patterns, whereas comprehensive multiyear datasets integrating regional and care-level differences remain scarce.

The predominance of β-hemolytic streptococci, *E. coli*, and *S. aureus* is consistent with previous studies identifying these organisms as major contributors to equine wound and abscess colonization and infection [5,6,26,27,28,29]. Research conducted in various countries, such as Germany, Great Britain, Switzerland, South Africa, and Canada, has consistently demonstrated that these pathogens are frequently isolated from equine wounds and abscesses, regardless of the geographical location [5,6,26,28,29].

While numerous studies have investigated ESBL-producing *E. coli* in horses, data on the overall prevalence of *E. coli* in equine wound infections remain limited. One study from Australia [30] reported *E. coli* in 12.2% of isolates obtained from equine wounds; however, the total number of isolates analyzed in that study (n = 565) was substantially lower than the present one (n = 4.464). Our finding of 3/4 GCR is of particular concern as it is commonly associated with ESBL production [31]. Similar to a recent study conducted in Germany on dogs and cats, we used resistance to 3/4 GCR (ceftiofur and cefquinome) as an indicator for highly resistant *E. coli*, although methodological and population differences limit direct comparison [32]. Resistance to ceftiofur and cefquinome in the present study was assessed phenotypically based on an antimicrobial sensitivity test. Molecular confirmation of the genes for ESBL production (e.g., by polymerase chain reaction) was not performed. Therefore, isolates were classified as 3/4 GCR *E. coli*, rather than ESBL-producing *E. coli*. These 3/4 GCR *E. coli* were identified in 106 cases (4.0% of positive samples), accounting for 15.2% of all *E. coli* isolates. Carriage rates of ESBL-producing *E. coli* have been reported in up to 7.3% of samples (209 samples in healthy, adult horses) in Canada [33], while studies from the United Kingdom documented rates of 6.3% of samples (645 samples) [34]. A German study detected ESBL-producing *E. coli* in 1.4% of nasal swabs (340 samples) and 10.1% of fecal samples from horses (318 samples) [32]. Other research on the fecal carriage of ESBL-producing *E. coli* reported prevalence rates ranging from 6.3% (of 650 samples) in community horses to 32.0% (of 37 samples) in hospitalized patients, although methodological differences between studies, including sample size, study design, and bacterial identification methods, limit direct comparability [34,35].

In this study, MRSA was identified in 208 cases (7.9% of positive samples), representing 35.9% of all *S. aureus* isolates. These results broadly align with previous reports from Canada, Switzerland, Austria, and Germany [6,11,36,37]. The prevalence observed in the present study was slightly lower than that of another investigation in which MRSA was detected in approximately 12.1% of wounds (58 swabs) [10]. Notably, this study did not determine the percentage of MRSA among all *S. aureus* isolates. A German study from 2014 reported MRSA in 41.3% of *S. aureus* isolates, although the sample size was smaller (604 swabs from horses within a 17-month sampling period) [36].

The detection of MRSA in the present dataset underscores its well-established clinical relevance in both veterinary and human medicine. The MRSA is widely recognized as a significant pathogen in companion animals, with notable public health implications due to its zoonotic potential [38]. Moreover, *S. aureus* remains a leading cause of nosocomial infections in humans, for example, hospital-acquired pneumonia, highlighting the importance of MRSA monitoring in both veterinary and human healthcare settings [39].

The prevalence of multidrug-resistant bacteria varies widely depending on the study design and methodology, as mentioned above. Nevertheless, numerous publications have underscored the increasing concern regarding rising AMR in equine medicine [40]. The most important factors for this development include the excessive use of antibiotics and the lack of effective alternative treatments.

The isolation of *Pseudomonas* spp. (6.1%), *Actinobacillus* spp. (3.9%), and *Acinetobacter* spp. (3.3% of all isolates, respectively) from equine wounds and abscesses is consistent with previous reports in veterinary medicine. A study conducted in Switzerland identified *Pseudomonas* spp. in 5.0% (378 swabs), *Acinetobacter* spp. in 3.4%, and *Actinobacillus equuli* in 3.7% of all isolates, respectively, indicating a broadly comparable distribution pattern, although direct comparison is limited by differences in the study design and sample size [6]. Slightly higher proportions were reported in Canada, where *Actinobacillus suis* and *Pasteurella* spp. each accounted for 9.4% of all isolates [29]. However, this study included only 83 isolates from 53 swabs [29], compared to 4464 isolates analyzed in the present study. A previous study from Germany reported a bacterial distribution at family level, identifying *Pasteurellaceae* (5.0%), *Moraxellaceae* (4.0%), and *Pseudomonaceae* (6.0%) among isolates from equine wounds [5].

*Pseudomonas* spp., *Actinobacillus* spp., and *Acinetobacter* spp. are well recognized in human medicine as common components of chronic or biofilm-associated wound infections [38,41,42]. Bacteria embedded within a biofilm matrix show reduced susceptibility to the host’s immune defense mechanisms and antimicrobial agents, which can lead to prolonged infection and delayed healing [43]. Although these pathogens have been consistently detected in all studies, they account for only a comparatively small percentage of isolates and, therefore, appear to play a less prominent role in equine wound and abscess infections.

### 4.1. Temporal Changes

Temporal changes from 2019 to 2023 were observed, with an increase in the groups other staphylococci, environmental bacteria, and other *Enterobacterales*, and a decline was observed for *Pseudomonas* spp. from 2021 to 2023 (Figure 2). These temporal changes may reflect a variety of factors, including changes in environmental exposure, diagnostic practices, or antimicrobial usage patterns; however, these mechanisms could not be assessed within the present dataset. To the best of our knowledge, published data addressing temporal trends in these bacterial groups in equine wound isolates are not available in the literature.

No statistically significant upward trend in 3/4 GCR *E. coli* prevalence was identified in this study. Previous research has reported an increasing resistance of *E. coli* isolates to ceftiofur between the periods 1999–2004 (9/123 isolates) and 2007–2012 (20/88 isolates), based on the analysis of 252 *E. coli* isolates [44]. Higher numbers or a longer time span would be needed to verify a trend toward a higher prevalence of 3/4 GCR *E. coli* over time, respectively. Furthermore, multidrug-resistant *E. coli* was detected more frequently among hospitalized horses, suggesting a potential association between hospital exposure and the emergence of resistant strains, although direct comparison with the present study is limited by differences in the study design and populations investigated [45]. However, direct comparison with previous studies is limited due to the scarcity of truly comparable multiyear retrospective datasets. Although some studies have reported annual distributions of bacterial species over multiple years [6,28], no formal statistical analyses were conducted. Temporal analyses should be interpreted with caution for bacterial groups with lower or fluctuating annual isolate numbers. Although negative binomial regression was applied to account for the variability in count data, some temporal patterns may reflect fluctuations inherent to routine diagnostic submissions.

### 4.2. Clinical vs. Primary Care Settings

To the best of our knowledge, this is the first study to investigate whether the type of care may influence the results of isolated bacteria in wounds and abscesses in horses. Significant differences were observed between samples submitted from C or P, with MRSA being notably more prevalent in C (Figure 3). Several factors may contribute to these differences. Samples submitted from P may include infections evaluated at an earlier stage of disease, whereas cases referred to C may more frequently involve complicated or non-resolving conditions. In addition, the referral status and previous therapeutic exposure may differ between these settings. However, detailed clinical information and antimicrobial treatment histories were not available in the present retrospective dataset. Therefore, the differences observed between C and P should be interpreted cautiously and no causal conclusions regarding underlying drivers can be drawn.

Structural characteristics of veterinary institutions may contribute to differences in AMR. Regarding small animal medicine, larger clinics have been shown to exhibit significantly higher rates of methicillin-resistant *Staphylococcus pseudintermedius* and reduced antimicrobial susceptibility compared to smaller practices [46]. These observations support the concept that institutional factors, such as a prolonged hospital stay, prior antimicrobial exposure, and referral-related case selection, may influence resistance dynamics, as suggested in previous studies [47,48]. While these data originate from canine populations, similar mechanisms may also be relevant in equine referral hospitals.

Hospitalization and prior antimicrobial exposure are well-established risk factors for MRSA colonization in horses [48]. One study reported colonization rates of up to 40.0% (of 30 horses) in long-term hospitalized horses [49]. Rates increase further when antimicrobials are administered [50]. A 2017 investigation in equine hospitals suggested that MRSA strains isolated in these settings may originate from human healthcare environments or derive from livestock-associated MRSA [51]. Although equine clinics are not considered major reservoirs for MRSA in the human population according to current evidence, zoonotic transmission remains a concern, particularly for individuals with occupational or close contact with horses [51]. While nasal colonization of veterinary personnel is relatively common in equine clinics, documented infections in this group remain infrequent [9].

β-hemolytic streptococci, *P. agglomerans*, and environmental bacteria were more frequently isolated from P samples (Figure 3). This difference was statistically significant. The high numbers of β-hemolytic streptococci in P may reflect the fact that there is a tendency of first treatment in P, resulting in high proportions of those bacteria. The increased detection of environmental bacteria as well as *P. agglomerans* in P samples may reflect multiple factors, including environmental exposure, case characteristics, or pre-analytical factors [52]. However, standardized sampling and handling conditions could not be assessed within the present dataset. No published studies currently directly compare microbiological findings between primary care and referral equine settings, limiting the interpretation of this observation.

A statistically significant upward trend over the five-year period in the group of other staphylococci was observed in P. This group in the present dataset comprised both coagulase-negative and -positive *Staphylococcus* spp. with limited known clinical relevance in equine wounds. To the best of our knowledge, the potential role of these organisms in equine wound microbiomes has not yet been systematically investigated.

### 4.3. Regional Differences

A previously published study identified regional differences in bacterial prevalence in small animals [53]. In order to examine the regional differences in bacterial isolates from horses, Germany was separated into three regions: the South, the Northwest, and the Northeast. This predefined regional grouping was based on the of equine sample submissions and major horse-holding areas within Germany, and was applied for descriptive and comparative purposes. The regional classification was not intended to account for all potential epidemiological determinants, such as horse density, human population density, or agricultural structure, which were not available within the dataset. Bacterial species not mentioned in the following section did not show any statistically significant regional differences. Both unadjusted and Bonferroni-adjusted *p*-values were reported to ensure transparency and account for multiple testing. While several associations did not remain statistically significant after correction, consistent trends across regions were observed, suggesting potential biological relevance despite reduced statistical significance.

The consistently high prevalence of β-hemolytic streptococci across all regions reinforces their major role in equine wound colonization and infection [1,6,15,29,54]. This group includes *Streptococcus equi* subsp. *equi*, the causative agent of strangles [55], which represents a host-restricted and highly contagious pathogen of horses primarily affecting the upper respiratory tract [56]. It also includes *Streptococcus equi* subsp. *zooepidemicus*, which is commonly regarded as a commensal organism of the equine oral cavity, pharynx, and respiratory tract, and frequently acts as an opportunistic pathogen [56]. Their widespread occurrence highlights their clinical relevance and supports the need for their routine consideration in empirical treatment strategies for equine wounds [57]. Despite the overall high prevalence, significant regional differences were observed, with a lower frequency in South Germany compared to the Northeast region (Figure 5). However, a direct comparison with the existing literature is only possible to a limited extent, as the bacterial groups used in this study were defined specifically for this analysis and do not fully correspond to the classifications described in earlier studies.

A significantly higher prevalence of *S. aureus* was observed in the Northwest. The MRSA was detected significantly more frequently in the Northwest and South, which was also shown for companion animals in a recent study [58]. Although both S. aureus and MRSA showed higher prevalence in the Northwest, MRSA additionally demonstrated a higher prevalence in the South. As MRSA represents a resistant subset of S. aureus, its regional distribution does not necessarily fully parallel the overall prevalence of S. aureus and may reflect additional resistance-related dynamics not assessable within the present dataset. Differences in the frequency of 3/4 GCR *E. coli* did not remain significant in any of the three regions after adjustment for multiple testing (Figure 5). However, elevated rates of 3/4 GCR *E. coli* were reported in the Northwest, particularly around Bremen, in a recent antimicrobial surveillance study from companion animals in Germany [53]. Although the studies mentioned originated from a different animal population, they support the concept that regional factors may influence the distribution of MRSA across veterinary settings [58]. The specific mechanisms underlying these regional differences remain unclear and may involve multiple environmental or management-related factors that could not be evaluated in the present study [59].

A tendency toward a higher prevalence of *Actinobacillus* spp. in the Northeast was observed; however, this difference did not remain significant after adjustment for multiple testing. The reasons underlying the regional tendency observed for *Actinobacillus* spp. remain uncertain. Multiple environmental, management, or population-related factors may contribute to these differences, although these variables were not available for analysis in the present dataset [47]. Although the Northeast has the lowest density of livestock holdings nationally, it is home to some of the largest and most industrialized animal production facilities in the country [59]. This structural pattern may influence bacterial transmission dynamics and partially explain any regional differences in pathogen distribution. However, the declining prevalence of *Actinobacillus* spp. in the Northwest suggests that local microbial dynamics may differ, although the underlying causes are probably multifactorial and remain poorly understood (Figure 5a,b).

Interestingly, some bacterial groups showed significant regional differences, including other *Enterobacterales* (higher prevalence in South than Northeast), *Pseudomonas* spp. (higher in Northeast than Northwest), and other Gram-negative bacteria (higher in South than Northwest) (Figure 5). However, no comparable data are currently available in the literature to support or explain these findings. Therefore, these observations should be interpreted with caution and may reflect environmental variation or sampling-related factors rather than consistent epidemiological patterns. Differences observed for environmental and spore-forming bacteria, as well as enterococci, did not remain significant after adjustment for multiple testing.

### 4.4. Clinical Implications

The presence of diverse bacterial species, including pathogenic and environmental organisms, highlights the polymicrobial nature of equine wounds [15]. This microbiological complexity presents challenges for clinical interpretation, particularly when assessing the pathogenic relevance of less frequently isolated tax, a such as *P. agglomerans* or spore-forming organisms [43,52]. These species are generally regarded as environmental opportunists—organisms not typically part of the normal equine microbiota but capable of acquiring clinical relevance under specific predisposing conditions, such as immunosuppression, extensive tissue injury, or inadequate wound management [43]. Detection of environmental organisms, including *Bacillus* spp. and *Pantoea agglomerans*, should, therefore, be interpreted with caution, as their presence may reflect true infection, colonization, or contamination, depending on the sampling procedure and clinical context.

When it comes to empirical antibiotic therapy in equine wound care, consideration should be given not only to the most likely pathogens, but also to regional variations and differences in the type of veterinary care provided with regard to bacterial isolates.

There is an increasing consensus that the prescribing of antibiotics in equine medicine needs to be reevaluated with a focus on more prudent use [60]. The detection of MRSA and 3/4 GCR *E. coli* in this study reinforces the importance of routine antimicrobial susceptibility testing and continuous resistance surveillance in equine practice [16,50,61].

Addressing AMR requires coordinated action that goes beyond clinical decision-making. The World Health Organization’s “One Health” framework emphasizes the interconnectedness of human, animal, and environmental health, and calls for collaborative strategies to combat AMR across sectors [31]. In alignment with this approach, the German Antimicrobial Resistance Strategy (DART 2030) outlines a comprehensive national and international action plan [62]. Building upon its 2008 predecessor, DART 2030 particularly emphasizes the need to strengthen research and development to more effectively address AMR and promote sustainable antimicrobial stewardship [62].

### 4.5. Limitations

The samples analyzed originated from routine diagnostic submissions to a commercial laboratory, reflecting real-world clinical practice. Based on the number of contributing practices and comparative data from national veterinary statistics, the dataset is estimated to cover approximately one-third of equine veterinary practices in Germany, supporting a broad representation of routine field submissions. Overall, missing data were rare relative to the size of the dataset, which is notable for a routine diagnostic laboratory setting.

Despite the robustness of the dataset and the large number of samples analyzed over a five-year period, several limitations of this study must be acknowledged. Detailed clinical histories, prior antimicrobial treatments, and wound characteristics (e.g., wound type, such as traumatic, surgical, or chronic, as well as the duration of infection or therapy) were not documented, limiting more detailed clinical associations and the interpretation of potential clinical and epidemiological risk factors. Demographic variables, including breed, age, and sex, were inconsistently reported and, therefore, not included in the analysis, although these factors may influence wound healing. Due to the anonymized nature of the laboratory dataset, information regarding repeated sampling from the same horse was not available. Therefore, the potential impact of duplicate submissions could not be assessed. However, according to expert assessment by the data provider, the frequency of duplicate submissions is considered to be low (less than 1% of isolates). The present study focused on bacterial isolates recovered from routine diagnostic submissions; therefore, culture-negative samples and factors potentially contributing to negative culture results, such as prior antimicrobial treatment, sampling quality, transport conditions, or low bacterial burden, could not be assessed.

Furthermore, anaerobic culture methods were not included in the present study. Therefore, the extent and bacterial spectrum of anaerobic involvement could not be assessed and may have contributed to an underestimation of bacterial diversity, particularly in abscesses and chronic wound infections.

This study was based on retrospective laboratory submissions; therefore, additional animal- and management-related variables as well as the detailed clinical context were unavailable, and unmeasured confounding may therefore have influenced some of the associations observed. Although the date of sample collection was available, the analyses focused on annual, regional, and care-setting patterns rather than detailed seasonal variation. Consequently, the findings should be interpreted primarily as descriptive patterns within routine diagnostic submissions rather than evidence of causal relationships. Furthermore, variations in sampling and transport conditions inherent to routine diagnostic submissions may have affected the viability and diversity of isolates. Finally, comparisons with previously published studies should be interpreted cautiously, as differences in sample size, study design, case selection, and bacterial identification methodologies may limit any direct comparability between datasets.

Despite these limitations, the present study provides a comprehensive overview of bacterial species distribution in equine wounds and abscesses based on a large, multiyear dataset. However, the results do not allow conclusions regarding clinical relevance, infection severity, or treatment outcome. Future studies integrating detailed clinical data and standardized sampling protocols are warranted to further refine the interpretation of these findings.

## 5. Conclusions

This five-year retrospective analysis of equine wound and abscesses, as well as bacterial species collected across Germany, confirms the predominance of β-hemolytic streptococci, *E. coli*, and *S. aureus*, consistent with findings published previously. The detection of MRSA and 3/4 GCR *E. coli* highlights the relevance of monitoring AMR in equine clinical practice and its broader implications within the One Health framework.

The regional and type of care differences observed regarding bacterial prevalence underscore the need for targeted surveillance systems and more nuanced antimicrobial stewardship strategies. Key limitations of the study include the absence of detailed clinical metadata and the lack of standardized sampling procedures.

Future prospective research should incorporate these components, alongside resistance profiling, to support evidence-based infection control and resistance mitigation in equine medicine.

## Figures and Tables

**Table 1:** Overview of the total number of samples from wounds and abscesses from horses from 2019 to 2023 separated into regions (South Germany, Northwest Germany and Northeast Germany) as well as into their type of veterinary care: P and C.

	2019	2020	2021	2022	2023	Total (over a Five-Year Period)
Samples (n)	625	534	521	547	617	2844
Positive samples percentage (%)	90.4	92.0	92.3	93.4	94.3	92.5
Bacterial isolates from samples (n)	926	817	829	881	1011	4464
Positive samples separated into regions						
South Germany (n)	279	244	195	197	266	1181
percentage (%)	49.4	49.7	40.5	38.6	45.7	44.9
Northwest Germany (n)	228	185	194	230	237	1074
percentage (%)	40.4	37.7	40.3	45.0	40.7	40.8
Northeast Germany (n)	58	62	92	82	79	373
percentage (%)	10.3	12.6	19.1	16.1	13.6	14.2
Positive samples separated into their type of veterinary care						
Primary Care (P) (n)	159	131	157	158	181	786
percentage (%)	28.1	26.7	32.6	30.9	31.1	29.9
Clinics (C) (n)	377	323	299	332	377	1708
percentage (%)	66.7	65.8	62.2	65	64.7	64.9
No Data (n)	29	37	25	21	24	136
percentage (%)	5.1	7.5	5.2	4.1	4.1	5.2

**Table 2:** Overview of bacterial groups and examples of representative species isolated from equine wounds and abscesses.

Group Name	Species
*Staphylococcus aureus*	*S. aureus*
*Escherichia coli*	*E. coli*
ß-hemolytic Streptococci	e.g., *Streptococcus* (*Strep.*) *equi* subsp. *equi*, *Strep. equi* subsp. *zooepidemicus*, *Strep. dysgalactiae*
*Pseudomonas* spp.	e.g., *Pseudomonas* (*P.*) *aeruginosa*, *P. koreensis*, *P. putida*
*Acinetobacter* spp.	e.g., *Acinetobacter* (*A.*) *baumannii*, *A. calcoaceticus*, *A. johnsonii*
Enterococci	*Enterococcus* spp.
*Actinobacillus* spp.	e.g., *Actinobacillus* (*A.*) *equuli*, *A. pleuropneumoniae*, *A. rossii*
*Pantoea agglomerans*	*Pantoea* (*P.*) *agglomerans*
Other *Enterobacterales*	*Enterobacter* spp., *Escherichia* spp., *Pseudoescherichia* spp., *Pantoea* spp., *Klebsiella* spp., *Proteus* spp., *Morganella* spp., *Rahnella* spp., *Leclercia* spp., *Lelliottia* spp., *Raoultella* spp., *Salmonella* spp., *Erwinia* spp., *Serratia* spp., *Buttiauxella* spp., *Citrobacter* spp., *Cronobacter* spp., *Ewingella* spp., *Hafnia* spp., *Kluyvera* spp., *Kosakonia* spp., *Providencia* spp.
Other Streptococci	*Streptococcus* spp.
Other Staphylococci	*Staphylococcus* spp.
Environmental bacteria	α-hemolytic Streptococci, *Actinomyces* spp., *Acranobacterium haemolyticum*, *Aerococcus* spp., *Corynebacterium* spp., *Lactococcus* spp. *Weisella* spp., *Micrococcus* spp., *Lactobacillus pentosus*, *Leuconostoc* spp., *Paenarthrobacter* spp., *Vagococcus fluvialis*, *Carnobacterium maltaromaticum*, *Pseudarthobacter* spp., *Rhodococcus* spp.
Spore-forming bacteria	*Bacillus* spp., *Paenibacillus* spp., *Exiguobacterium* spp., *Kurthia* spp.
Other Gram-negative bacteria	*Aeromonas* spp., *Myroides* spp., *Pasteurella* spp., *Stenotrophomonas* spp., *Moraxella* spp. *Advenella* spp., *Alcaligenes* spp., *Burkholderia* spp., *Comamonas* spp., *Delftia* spp., *Empedobacter* spp., *Frederiksenia* spp., *Haemophilus* spp., *Massilia* spp., *Myroides* spp., *Ochrobactrum* spp., *Wohlfahrtiimonas* spp., *Bergeriella* spp., *Neisseria* spp., *Psychrobacter* spp.

**Table 3:** Overview of the sample origin, either C or P, between 2019 and 2023 and the percentage of swabs submitted by C or P from which bacterial isolates were obtained. Percentages represent the proportion of positive samples within each sampling facility, C or P, compared to the total number of samples submitted from that category. Only the results of positive swabs are shown, respectively.

	Total (N)	Origin: C (%)	Origin: P (%)	Positive Swabs from C (%)	Positive Swabs from P (%)
2019	565	66.7	28.1	88.7	95.2
2020	491	65.8	26.7	90.2	95.6
2021	481	62.2	32.6	91.4	92.9
2022	511	65.0	30.9	91.2	97.5
2023	582	64.7	31.1	93.1	96.8
Total	2630	64.9	29.9	90.9	95.6

## Data Availability

The data presented in this study are available on request from the corresponding author.
